# Medicinal Plants and Their Bacterial Microbiota: A Review on Antimicrobial Compounds Production for Plant and Human Health

**DOI:** 10.3390/pathogens10020106

**Published:** 2021-01-22

**Authors:** Lara Mitia Castronovo, Alberto Vassallo, Alessio Mengoni, Elisangela Miceli, Patrizia Bogani, Fabio Firenzuoli, Renato Fani, Valentina Maggini

**Affiliations:** 1 Department of Biology, University of Florence, 50019 Florence, Italy; laramitia.castronovo@unifi.it (L.M.C.); alberto.vassallo@unifi.it (A.V.); alessio.mengoni@unifi.it (A.M.); micelielisangela@gmail.com (E.M.); patrizia.bogani@unifi.it (P.B.); 2 CERFIT, Research and Innovation Center in Phytotherapy and Integrated Medicine, Tuscany Region, Careggi University Hospital, 50141 Florence, Italy; fabio.firenzuoli@unifi.it

**Keywords:** medicinal plants, plant microbiota, bacterial endophytes, antimicrobials

## Abstract

Medicinal plants (MPs) have been used since antiquity in traditional and popular medicine, and they represent a very important source of bioactive molecules, including antibiotic, antiviral, and antifungal molecules. Such compounds are often of plant origin, but in some cases, an origin or a modification from plant microbiota has been shown. Actually, the research continues to report the production of bioactive molecules by plants, but the role of plant–endophytic interaction is emerging. Classic examples are mainly concerned with fungal endophytes; however, it has been recently shown that bacterial endophytes can also play an important role in influencing the plant metabolism related to the synthesis of bioactive compounds. In spite of this, a deep investigation on the power of MP bacterial endophytes is lacking. Here, an overview of the studies on MP bacterial microbiota and its role in the production of plant antimicrobial compounds contributing to prime host defense system and representing a huge resource for biotech and therapeutic applications is provided.

## 1. Introduction to Medicinal Plants

Medicinal plants (MPs) are used in long-established practices of traditional medicines in many countries. Traditional medicine is the total sum of the practices based on the theories, beliefs, and experiences of different cultures and eras. It is often inexplicable and used to maintain and improve health, as well as in the prevention, diagnosis, and treatment of illnesses [1]. The World Health Organization established definitive guidelines regarding the methodology of clinical research and the effectiveness appraisal of traditional medicine. Over time, especially in relation to ethnobotanical and ethnopharmacological studies, preparations based on MPs have become an integral part of mainstream medicine, and nowadays, they are used as a source of chemical substances, either directly (e.g., atropine, morphine, etc.), or for chemo-pharmaceutical hemi synthesis (e.g., acetylsalicylic acid, paclitaxel, etc.). Additionally, MP extracts are exploited because of their phytocomplex when it is demonstrated that a precise pool of compounds has a different and better pharmacological effect than the single constituents. For example, it is well known that the antidepressant effect of St. John’s wort (*Hypericum perforatum*) only occurs in the presence of the phytocomplex standardized in flavonoids, hypericins, and hyperforins [2]. Single active substances are often used in medicine, even for serious pathologies, such as in the case of taxol for neoplastic pathologies, artemisinin as an antimalarial agent, and morphine as an analgesic. On the contrary, extracts containing the MP phytocomplex are more often used in the prevention and treatment of numerous diseases of mild and medium severity: rheumatic diseases with inflammatory nature (*Boswellia serrata*, *Harpagophytum procumbens*), cardiovascular diseases (*Crataegus monogyna*), metabolic diseases (red fermented rice), neuro-psychic disorders (*Hypericum perforatum*, *Rhodiola rosea*), urinary genital disorders (*Serenoa repens*), digestive system disorders, such as irritable colon (essential oil of *Mentha piperita*), and recurrent infections of the ENT (ear, nose, and throat) and the bronchopulmonary system (*Eucalyptus globulus*, *Echinacea purpurea*).

Research in this field is based on the knowledge of different scientific disciplines (i.e., botany, plant biology, phytochemistry, pharmacology, toxicology, pharmacokinetic studies, pharmacovigilance, and clinical trials), with the final goal being the evaluation of the quality, efficacy, and safety of herbal medicines, as requested by many regulatory authorities worldwide [3]. In the last decades, research has increased to discover MPs to be used synergistically with synthetic drugs, reducing side effects (e.g., *Astragalus membranaceus* on neutropenia, *Zingiber officinale* on chemotherapy-induced nausea, *Cannabis sativa* to reduce the dosage of opioids, and caffeine to reduce the dosage of nonsteroidal anti-inflammatory drugs against pain). Moreover, MPs can also play an important role against the insurgence of antibiotic resistance both directly for their antimicrobial activities (e.g., antibacterial, antiviral, antifungal, and antiparasitic ones) and indirectly by reducing the resistance against antibiotics.

When used in medical therapy, it is important to consider that MPs are complex and dynamic systems. Thus, their chemical composition varies depending on several factors, such as botanical species, genetically determined chemotypes, anatomical part of the plant used (e.g., seed, flower, root, leaf, etc.), storage, sun exposure, humidity, type of ground, time of harvesting, and geographic area. In addition, biogenic factors, such as the bacterial and fungal endophytes associated with various parts of the plant, can influence their chemical composition. In recent years, the study and research of the numerous interactions occurring between MPs and endophytes are revolutionizing our knowledge of plant biology, with completely unexpected and very large application perspectives: the possibility of modulating, amplifying, or interfering in the biosynthesis of phytoconstituents (e.g., terpenes, polyphenols, alkamides, etc.), but also to directly engineer the synthesis of new molecules, for example with antibiotic activity.

There are numerous MPs with documented antimicrobial activity in vitro and in vivo, including examples from Traditional Chinese or Ayurvedic Medicine. The novelty of most recent research is related to the availability of clinical tests through clinical trials conducted with methodological rigor. Indeed, although being active in vitro, many substances are not suitable for clinical practice due to poor bioavailability or direct toxic effects on the human body. Therefore, all preclinical research must also be supported by clinical pharmacological studies and exploit new pharmaceutical technologies, such as nano-formulations of active substances.

The purpose of this review is to highlight the role of the bacterial microbiota of MPs in the production of plant antimicrobial compounds that, contributing to plant health and plant therapeutic properties, represent a huge resource for biotech and therapeutic applications.

### 1.1. MPs and Antimicrobial Power

Evidence of MP clinical efficacy having antimicrobial activity is scarce, despite positive preclinical results. However, current results are promising and confirm that the traced path is the right one. In some cases, MPs have been shown to harbor endophytes potentially involved in the biosynthesis of phytoconstituents and/or to be able to synthesize bioactive compounds.

Among MPs adopted in clinical studies, an example is the plant neem (*Azadirachta indica*), which is an evergreen tree of the tropics and sub-tropics, native to the Indian subcontinent, with a demonstrated ethnomedicinal value and importance in agriculture as well as in the pharmaceutical industry [4]. In a clinical study, the impact of neem-containing mouthwash on plaque and gingivitis was investigated, demonstrating that it can be used for maintaining oral hygiene and that it might have a better impact in the prevention of oral diseases, as it is both cost-effective and easily available [5]. So far, more than 400 compounds have been isolated from different parts of neem, including important bioactive secondary metabolites, and more than 30 compounds have been isolated from neem endophytes [4].

Artemisinin (obtained from *Artemisia annua* L.) is already registered as a drug used for the treatment of malaria [6], it is effective against all species of *Plasmodium,* and it is particularly useful in case of infections by chloroquine-resistant and multidrug-resistant parasites. Artemisinin works by killing *Plasmodium* at the schizont stage. Unfortunately, the yield of this antimalarial drug is low in *A. annua* plants (0.01–1.1%), resulting in low availability and high cost. In a recent study, the effects of neem root endophytes on the artemisinin production in *A. annua* plants were evaluated [7,8]. The concentration of artemisinin and the expression profiles of artemisinin biosynthetic genes were significantly higher in the treated plants, suggesting the potential use of endophytes for greater productivity of *A. annua* and its sustainable agriculture.

The antimicrobial activity of *Origanum vulgare* essential oils (EOs) has been well studied in veterinary medicine, for example, in *Malassezia pachydermatis*, the causal agent of dog dermatitis. The treatment with a 0.5% oily preparation achieved a good clinical outcome without relapses in comparison to the treatment with ketoconazole [9].

Previous clinical investigations suggest the use of extracts from the root of *Pelargonium sidoides* for the therapy of uncomplicated acute upper airway inflammations due to its strong antimicrobial and immunomodulatory effect [10]. The therapy with *P. sidoides* significantly decreases the frequency of patients with positive cultures of *Streptococcus pneumoniae*, *Haemophilus influenzae*, and *Moraxella catarrhalis*.

Furthermore, berberine, a plant alkaloid isolated from many MPs, has shown antimicrobial activity against selected oral pathogens, and it is more effective than saline as an endodontic irrigant against selected endodontic pathogens [11]. A bioinformatic study has proved that berberine content in *Coptis teeta* roots is positively correlated with total nitrogen, total phosphorus, total potassium, and available potassium in the rhizosphere soil [12]. In particular, the berberine content in roots has been positively correlated with *Microbacterium* and *norank_f_7B-8*, while total soil potassium is positively correlated with *Microbacterium* and *Burkholderia*-*Paraburkholderia* in roots.

*Echinacea* is a medicinal plant used in therapy and prevention of infectious diseases of the upper respiratory tract, also recently studied for the presence of bacterial endophytes influencing the biosynthesis of alkamides and caffeic acid derivatives [13,14]. The combined use of *Echinacea* with azithromycin has produced favorable outcomes in comparison to the use of azithromycin alone in pediatric patients with recurrent tonsillitis [15]. Moreover, in a clinical trial, a combination of *E. purpurea* has been able to alleviate the exacerbation symptoms caused by upper respiratory tract infections, a known frequent cause of exacerbations of chronic-obstructive pulmonary diseases [16].

It has been reported that *Leptospermum scoparium* (Mānuka), a New Zealand native medicinal plant that is well-studied for the bioactivity of its bacterial endophytes, produces EOs with antimicrobial properties [17]. Mānuka honey has revealed a significant antibiofilm activity in vitro and in vivo against *Staphylococcus aureus*, methicillin-resistant *S. aureus*, and *Pseudomonas aeruginosa*, and, in a recent phase 1 clinical trial, its safety and preliminary efficacy were investigated in recalcitrant chronic rhinosinusitis, resulting as a safe and effective as antibiotics [18].

### 1.2. MPs Bacterial Endophytes

**Figure 1:**
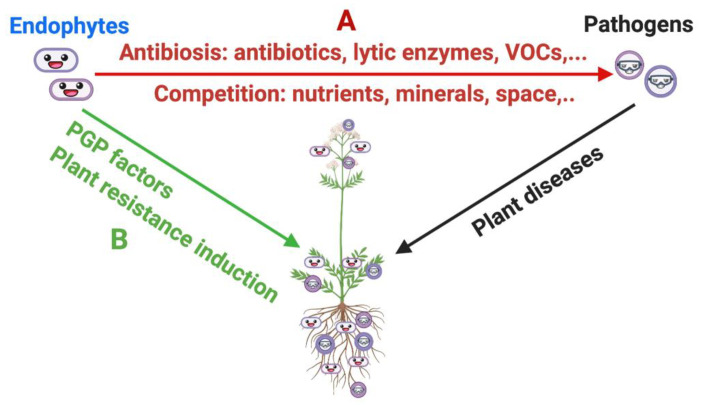
Actions of bacterial endophytes on host plant health. (**A**): Direct effects on pathogens, from the competition at niche level (colonization) to the production of antimicrobial compounds; (**B**): Indirect effects on pathogens, induction of host defense response, and promotion of plant growth. Figure created using BioRender (https://biorender.com/).

The use of massive DNA sequencing technologies in the last ten years has promoted a large advancement in the knowledge of plant-associated microbiota. Such intimate association of microorganisms has broad implications for plant nutrient assimilation, growth, stress tolerance, and health status, as well as secondary metabolite production [19] (Figure 1).

Many studies aim to understand the dynamics of such intimate association, its evolutionary significance, and the possibility to manipulate it to improve the host status [20,21]. Now, several works have been performed on the effect that the plant microbiota has on phenotypic and physiological features of the host plant. For instance, metal hyperaccumulating plants are shown to harbor a (possibly) co-evolved microbiota [22,23], and an important role in microbial-assisted phytoremediation has been recognized [24]. Furthermore, concerning bacterial microbiota associated with MPs, the comprehension of the forces driving the composition and structure of such microbial population(s) acquires even higher importance since it has been demonstrated that the plant microbiota plays a relevant role in the production of the plant secondary metabolites with therapeutic effects. As cited above, for example, the plant–endophyte interaction strongly acts on the secondary metabolism of *E. purpurea* [13,14].

Despite the importance that the microbiota exerts on MP properties, little is known about the forces shaping it [25]. It has been reported that the composition and structure of bacterial microbiota are related to both biotic and abiotic factors [26]. For example, continuous cropping and plant disease can influence the structure of plant microbiota, reducing rhizospheric and root bacterial diversity, as reported for *Panax notoginseng* [27]. Moreover, the soil also is strongly affecting microbiota associated with MPs. A work where three MPs are cultivated in a desert farm [28] has shown that the microbiota associated with the MPs, *Matricaria chamomilla* L., *Calendula officinalis* L., and *Solanum distichum* Schumach. and Thonn. is different from the microbiota associated with plants from other soils (i.e., desert soil uninfluenced by anthropogenic activity and humidity). The distinctive and intriguing evidence emerging from this study is that plants tend to select from soil those microbial populations able to provide them with better fitness, such as those belonging to the *Firmicutes* phylum, characterized by high resistance in arid environments. This kind of selection has been demonstrated, for example, in the case of fungal endophytes from *Leymus mollis* [29].

The biotic interactions are believed to be the most important factor for the maintenance of the stability within a microbiota [30], and at least three different scenarios might be depicted to explain how endophytes that colonize MPs are selected. In the first case, secondary metabolites present in plant tissues could determine a selective action on the composition of the plant microbiota [31]. Examples of secondary metabolites that could exert this selective effect are those forming EOs. For instance, in *O. vulgare* [32], a correlation between a fraction of the endophytic microbiota (namely lactic acid bacteria) and the EO content has been found, suggesting that the colonization within plant niches may be regulated by mechanisms linked to the synthesis of secondary metabolites.

Similar evidence emerges from the analysis of bacterial microbiota associated with two phylogenetically close MPs, *Thymus vulgaris* and *T. citriodorus* [33]. The plant microbiota has shown different tolerance levels to the plant EOs, which may determine the selection of certain bacterial taxa, and the total microbiota in the rhizosphere and plant tissues correlates with the different content in EOs among plant compartments and plant species.

A second scenario implies the presence of specific endophytes selected based on their metabolic abilities that best fit the plant environment. As reviewed elsewhere [34], cases in which (i) bacterial endophytes provide the host plant with siderophores, or (ii) endophytes are able to trigger a cross-talk modulating the production of phytohormones, or (iii) bacteria increase plant tolerance to stresses (e.g., drought) have been reported. Moreover, it has been demonstrated that the biosynthesis of the anticancer and cytotoxic agent maytansine in *Maytenus serrata* is shared among this plant and its endophytic bacterial community. Indeed, the host produces the starter unit of the biosynthetic pathways, and the endophytes perform the remaining biosynthetic steps leading to the final compound [35].

Lastly, but not least, endophytes could be selected in relation to their antimicrobial resistance phenotype as a response to antimicrobial molecules produced by microorganisms in the same ecological niche [31]. This hypothesis has been suggested in the case of the three MPs, *E. purpurea* and *E. angustifolia* [13,31,36], and *O. vulgare* [37]. Here, different plant compartments were characterized by different antimicrobial resistance patterns and antagonistic interactions. In particular, the stem/leaf compartment showed the presence of bacterial endophytes with the lowest degree of antibiotic resistance and the highest sensitivity towards the inhibition exerted by bacteria isolated from rhizospheric soil and roots. On the contrary, the rhizospheric soil and root compartment evidenced bacterial endophytes with higher antibiotic resistance levels and lower sensitivity towards the inhibition in comparison to the stem/leaf tissues. These aspects depict the rhizospheric and root compartments as more competitive environments, with respect to the stem/leaf one.

The above three different scenarios might not be mutually exclusive, and it is likely that they represent different aspects and/or functional moments of the complex relationship between the host and its microbiota.

Interestingly, the scenario of bacterial endophytes as determinants of the structure of their own microbiota by means of antimicrobial molecules depicts the plant microbiota as an important resource of relevant biotechnological molecules that could be used to solve health-associated problems, such as those related to antimicrobial resistance. Indeed, the potential of *Echinacea*-derived bacteria as sources of antimicrobial molecules has already begun to be explored, showing important inhibitory action towards antibiotic-resistant opportunistic pathogens from *Burkholderia cepacia* complex (Bcc) [38,39], determinants of severe infections in immunocompromised patients.

#### Experimental Approaches for the Study of MP-Bacteria Interaction

The study of the endophyte bacterial communities has surely taken advantage of the development of novel experimental approaches based on high throughput sequencing of DNA. Indeed, their spread and increased affordability have set these techniques as a standard to study bacterial communities. The most widespread approach is based on sequencing of 16S rRNA coding gene, and different pipelines for data analysis have been developed for this purpose. In this way, it has been possible to become knowledgeable about the whole bacteria community, regardless of strain-specific cultivability. However, this experimental approach does not allow the identification of bacterial isolates to the species level, and so, in the best scenario, conclusions concern taxonomic composition only at the genus level. For such reasons, culture-dependent procedures cannot usually be omitted because bacterial isolation allows specific investigations at the species-level, besides providing the opportunity to uncover traits of interest (e.g., production of specific metabolites, resistance against antibiotics, etc.) both for biotechnological purposes and for studying the physiology regarding interaction with the host. Although cultivation-dependent approaches are surely essential, their main drawback is related to the use of media whose composition is not always able to sustain the growth of all different taxa. This may be due, for example, to a different balance between nutrients in comparison to that found in plant tissues and/or to the missed establishment of bacterial consortia able to support the growth of all species. At this regard, it seems promising the use of in situ *similis* culturing aiming to mimic the complex environmental composition observed in plant tissues [40]. Indeed, Nemr and colleagues have demonstrated that using either leaves as solid substrate or media obtained upon infusion of leaves, the diversity of isolated bacteria results increased in comparison to the use of the standard medium R2A as control.

However, it should be underlined that current approaches usually underestimate the actual distribution of microorganisms since the whole plant compartments are usually studied, without considering single microenvironments. As highlighted in [41], different niches can be defined even in a single leaf, where several features specifically characterizing the upper and lower surfaces. The proximity to veins, etc., could influence and specifically select the microbial population.

Several MPs have been analyzed for the presence and composition of bacterial microbiota associated with their different tissues. Common evidence that emerges is that different plant compartments exhibit specific bacterial microbiota. For example, the analysis of cultivable endophytic bacteria associated with different tissues of *Aloe vera* (root, stem, and leaf) [42] reveals the presence of 13 genera in different percentages in each compartment, showing the presence of specific bacterial microbiota. Analogously, other works concerning different MPs tissues have been conducted with *Lavandula angustifolia* Mill. [43], *E. purpurea* L. Moench, and *E. angustifolia* (DC.) Hell [44]. The extraction of culturable endophytic bacteria from different tissues (stem, leaf, root) of *L. angustifolia* [43] evidences the presence of 11 bacterial genera, with an extremely different distribution in plant tissues. In the case of *E. purpurea* and *E. angustifolia* grown very close to each other in the same soil [44], there are no common bacterial strains among the three compartments (root, leaf, and rhizosphere) of a single plant. Furthermore, the two plant species shared only 23 out of 380 random amplification of polymorphic DNA (RAPD) haplotypes, showing a high level of specificity even between the two plant species.

## 2. Antimicrobial Power of the MPs Bacterial Microbiota

### 2.1. Studies on Antimicrobial Activity of MP Bacterial Endophytes

Among endophytes, fungi are the most commonly isolated and studied microorganisms, and, in this context, taxol is a paradigmatic example of endophytic bioactive potential [45]. Despite the copious amount of papers about the interaction between plants and fungal endophytes available in the literature, studies concerning bacteria still only number a few, and they mainly regard the identification of taxa and the characterization of strains with interesting features, such as the production of bioactive compounds and hydrolytic enzymes. Moreover, papers aiming to shed light on the in vivo molecular mechanisms involving bacteria and hosts are even less.

In our attempt to summarize current results, we interrogated the database Pubmed using the following query: [(medicinal plant*) AND (endophyt*) AND (bacteria)], limiting the research to items published in English between 2015 and 2020. Our research retrieved a total of 350 results (updated to May 2020): without considering article types other than original papers (e.g., reviews and genome announcements), discarding those regarding exclusively fungi and those not about MPs, only 126 items have met our selective criteria (Table S1).

Most of them were about MPs from Asia (87), mainly China (51) and India (21), followed by Europe (9), Africa (8), South America (6), Oceania (3), and North America (2) (in the remaining 11 the geographic area was not defined). Eighty-five of them used terms clearly denoting the use of the described plant species as part of traditional medicines (e.g., Traditional Chinese Medicine and Ayurveda) and/or folk medicine. One hundred and fifteen exclusively studied bacteria, while 9 regarded both bacterial and fungal endophytes. In seven works, bacterial communities were characterized through both culture-dependent and culture-independent approaches. On the contrary, 113 considered only cultivable bacteria, and six lacked isolation of strains (i.e., they were based only on next-generation sequencing of metagenomes). In about half of the cases (56 out of 126), just a single plant compartment was characterized. Only 24 articles included experiments aimed at studying the molecular aspects of the bacteria–host plant interaction and/or in vivo approaches to investigate the influence of bacteria in plant growth and metabolism. In this regard, they studied, for example, the effect of different bacterial inoculants on seed germination, plant growth, and resistance against phytopathogens, and the comparison of metabolite profiles between inoculated and not inoculated plants.

Analyses on the bioactivity of MP microbiota have evidenced many bioactive molecules with different functions, revealing the presence of important antibiotic compounds already characterized and others never described before. For instance, a *Bacillus* strain isolated from stems of the Indian traditional medicinal plant *Bacopa monnieri* L. showed an inhibitory effect on the growth of phytopathogenic fungi *Rhizoctonia* sp., *Sclerotium* sp., and *Phytophtora* sp. [46]. In addition, this bacterium was able to inhibit pathogenic targets, such as *Escherichia coli*, *Salmonella enterica* Tiphy, *B. subtilis*, *S. aureus*, and *Klebsiella pneumoniae*. Interestingly, important antibiotics, such as surfactin, iturin, and fengycin, were found among the bioactive fractions of the bacterial strain extracts. A broad-spectrum antimicrobial activity was highlighted for another *Bacillus* endophytic strain, isolated from an ancient oriental medicinal plant, *Andrographis paniculata* Nees [47]. This strain was able to inhibit bacterial pathogens, such as *B. subtilis*, *B. cereus*, *Vibrio parahaemolyticus*, *Aeromonas caviae*, *Proteus vulgaris*, and *P. aeruginosa*. The analysis of the single extracts showed the presence of three different anti-infective metabolites, and one of these was an anthracene derivative. A novel group of bioactive substances, named munumbicins, was isolated from *Streptomyces* sp. NRRL 30562, extracted from the stem tissue of the medicinal plant *Kennedia nigriscans* [48]. Munumbicins are active against plant–pathogenic fungi and human–pathogenic bacteria, comprising antibiotic-resistant strains. Munumbicin B showed to be active against multiple-drug resistant *Mycobacterium tuberculosis*. Remarkably, each munumbicin molecule was active against *Plasmodium falciparum*, the most pathogenic plasmodium causing malaria. Bacterial isolates from the medicinal plant *E. purpurea* showed inhibitory activity against human opportunistic pathogens of Bcc, which cause severe infections in immunocompromised patients [38]. More than 97% of the tested strains exhibited inhibition on the growth of Bcc strains, with either environmental or clinical origin. In particular, bacteria associated with the root compartment exhibited the highest degree of inhibition in comparison to bacteria isolated from the other plant compartments. The bioactivity of *E. purpurea* associated bacterial strains was also evidenced in the plant rhizosphere. Indeed, the strain *Rheinheimera* sp. EpRS3 exhibited complete inhibition of all the analyzed *B. multivorans* and *B. cenocepacia* strains and other clinically relevant human pathogens, such as *Acinetobacter baumannii* N50 and *A. baumannii* YMCR 363 [39].

Actinobacterial endophytic strains, mainly *Streptomyces*, from the Brazilian medicinal plant *Lychnophora ericoides* demonstrated activity not only against bacteria and yeast but also against human cancer cell lines, showing its cytotoxic potential [49]. Very high cytotoxic activity was demonstrated for 39% of the tested extracts versus different cancer cell lines. The antioxidant potential of endophytic bacteria was evidenced by *Paenibacillus polymyxa* EJS-3 isolated from the Chinese medicinal plant *Stemona japonica*. The exopolysaccharide (EPS) of the strain was synthesized in vitro, and both crude and purified EPS demonstrated strong scavenging activity on superoxide and hydroxyl radicals. The wide spectrum of the activity of endophytes-derived molecules extends to anti-inflammatory effects. Carbazole derivatives obtained from the endophytic *Streptomyces* sp. LJK 109 [50] suppressed macrophage production of the inflammatory mediators NO, PGE2, TNF-α, IL-1β, IL-6, IL-10 in a dose-dependent manner.

The described examples highlight the huge potentiality of MP-associated bacteria to produce bioactive molecules with a wide range of applications and underline how MP microbiota is a good source to isolate biocontrol agents for both human and plant pathogens.

### 2.2. Studies on the Role of MP-Endophyte Interaction in the Production of Bioactive Molecules: Focus on Antimicrobial Compounds

In planta production of bioactive compounds can depend on the yield of biomass and/or alteration of specific metabolic pathways, which can be mutually influenced or even shared between the host plant and its endophytes [51]. Although, in general opinion, the role of bacteria in MP fitness is considered of great relevance, current literature does not provide extensive investigations about this topic. Indeed, as shown above, papers are often limited to the study of microbial communities without a systematic investigation of the molecular dynamics between endophytes and host. Moreover, bacteria associated with MPs have been lesser-explored than their fungal counterpart. However, they have been recently described as able to influence the synthesis of secondary metabolites with therapeutic properties [13,52]. Bacteria can enhance the production of bioactive compounds either directly through the tuning of biosynthetic gene expression or indirectly by improving the biomass yield and general plant wealth (for example, through phosphate solubilization, nitrogen fixation, and indole acetic acid production-IAA), as demonstrated in the case of alkaloids in *Catharantus roseus*, *Papaver somniferum*, and *Withania somnifera* [53,54,55]. Analogously, it has been demonstrated that *Lycoris radiata,* inoculated with endophytic bacteria isolated from different plant districts, has an increased accumulation of alkaloid compounds [56]. Indeed, these endophytes are able to benefit the primary metabolism through either the production of IAA or the fixation of nitrogen (as demonstrated by the presence of the *nifH* gene), thus providing increased starting substrates (e.g., amino acids) for the synthesis of alkaloids. Interestingly, bacteria belonging to different genera increase the concentration of different alkaloids, indicating the presence of diverse metabolic routes. In addition, bacterial endophytes can also protect the MPs from phytopathogens through antagonistic activities [57] and can promote plant growth, alleviating oxidative stress by scavenging the reactive-oxygen species, as the case of a *Streptomyces* strain isolated from *Mirabilis jalapa* [58]. Here, examples of how endophytic bacteria are able to affect the biosynthesis of secondary metabolites positively are reported. For example, an endophytic *Pseudonocardia* strain isolated from *A. annua* tissues, when inoculated in *A. annua* seedling, increased accumulation of artemisinin, as a consequence of the up-regulation of key biosynthetic genes (i.e., cytochrome P450 monooxygenase and reductase) [7,8]. In other cases, the inoculation of bacterial endophytes, previously isolated by the roots of *W. somnifera*, has increased the withanolide content in plant tissue during *Alternaria alternata* pathogenicity and upregulated the genes involved in withanolide biosynthetic pathways in both leaves and roots [54,59]. In another case, the bacterial endophytic *P. fluorescens* strain was able to enhance the accumulation of EOs in *Atractylodes lancea* through molecular signaling involving hydrogen peroxide, gibberellin, ethylene, and abscisic acid [60]. The same authors have also demonstrated that this strain of *P. fluorescens* induces a high production of medicinal sesquiterpenoid in *A. lancea* [61]. In particular, the authors report the bacterial synthesis of IAA with the consequent development of plant roots and their increasing capacity to assimilate essential carbohydrates for terpenoid hydrocarbons biosynthesis. In *Limonium sinense*, the inoculation with a *Glutamicibacter* endophyte increased plant growth and resistance against NaCl stress, and it was associated with an upregulation of genes involved in phenylpropanoid and flavonoid biosynthesis [62]. Both plant biomass and antioxidant content could be improved in *Achyranthes aspera* L. through the inoculation of a *P. aeruginosa* strain [63]. Indeed, germ-free plantlets treated with this endophyte showed higher concentrations of nitrogen, phosphorus, and potassium, and they had longer shoots and roots, and leaves had an augmented surface in comparison to the control. As reported above, endophytes can indirectly exert a positive effect on plant metabolite modulation by improving plant biomass. An example has been reported in the case of the New Zealand medicinal plant *Pseudowintera colorata* and its endophytic *Actinobacteria* [64]. In this context, it is noteworthy to underline that, besides promoting the growth of medicinal plants from whom they were isolated, there are examples of how endophytes might be used to improve the yield of commercial crops, as demonstrated in the case of *Medicago sativa* (alfalfa) and *Triticum aestivum* (wheat) [65]. Indeed, in these plants, both germination percentage and biomass yield increased upon inoculation with *Rahnella*, *Rouxiella*, and *Serratia* strains.

## 3. Biotechnological and Therapeutical Applications

Mutualistic interaction between the host plant and endophytes chemically supports the reaction to environmental stresses: metabolic versatility and biodiversity make the endophytes a great source of bioactive molecules [46]. In fact, endophytic biosynthetic adaptation leads to a high metabolite diversity to be explored for the discovery of drugs against plant and human pathogens.

The well-known example of the endophytic fungi from *Taxus* spp. and the related advance of industrial taxol production resemble the challenge of biotechnological approaches (e.g., in vitro manipulation and genome mining) to enhance the productivity of a bioactive product since taxol productivity is remarkably affected by endophyte sub-culturing and storage [66]. Hence, the identification of promising microorganisms must undoubtedly proceed for the development of sustainable exploitation methods in clinical, industrial, and agriculture fields. To this aim, several works highlight the opportunity offered by the analysis of plant–endophytes interaction to discover new molecules with high therapeutic potential against human pathogens [67,68,69]. Moreover, there are numerous examples of the use of endophyte consortia, or combinations with other microorganisms and/or inhibitors to fight dangerous phytopathogens through an enhancing effect on plant growth [70,71,72,73,74,75]. Further development of in vitro plant–endophytes models is, therefore, needed and desirable to shed light on the exploitation of new resources in these fields.

### 3.1. Biological Control of Plant Pathogens

One of the tools to control the development of plant diseases in plant pathology is the use of antimicrobial compounds produced by microbial antagonists. This strategy can be referred to as “biocontrol,” and microorganisms that suppress the causal agents of plant diseases are defined as “biological control agents” (BCA) [76], together with macroorganisms, chemical mediators, and natural substances [77]. Among microbial antagonists, endophytes—mainly fungal but also bacterial ones—play an important role in disease control [78,79,80,81]. Both of them contribute to biological control through different modes of action, from the competition at niche level (colonization) through the production of antimicrobial compounds (Figure 1A) to the induction of host defense response [79,81,82], thus promoting plant growth and health (Figure 1B). As reported before, a clear example of the microbiome importance to survive in environmentally adverse conditions is provided by results of Köberl et al. [28], showing the capability of three MPs, *M. chamomilla* L., *C. officinalis* L., and *S. distichum,* to grow on desert soil rich in fungal phytopathogens thanks to the presence of endophytic bacterial communities (especially *Bacillus* strains) displaying antagonistic activity against soil-borne phytopathogens. Other studies performed on major MPs (*Cymbopogon citratus*, *Majorana hortensis*, *Marrubium vulgare*, *M. chamomilla*, *Melilotus officinalis*, *Melissa officinalis*, *Ocimum basilicum*, *O. syriacum*, *Quisqualis indica*, *Solidago virgaurea*, *T. vulgaris* [83]; *Atropa belladonna*, *Cassia angustifolia*, *C. roseus*, *Dioscorea* spp., *Glycyrrhiza glabra*, *Hyoscyamus niger*, *P. ginseng*, *P. somniferum*, *Plantago major*, *Plectranthus barbatus*, *Podophyllum peltatum*, *Rauwolfia serpentina*, *W. somnifera* [84]) stated that endophytic plant growth-promoting *Rhizobacteria* (PGPR) belonging to *Bacillus*, *Azotobacter*, *P. fluorescens,* and *Actinomycetes* showed activity against fungal, bacterial, viral, or nematode causal agents of different MP diseases. Among PGRP, some showed the production of volatile compounds (VOCs), such as the hydrogen cyanide (HCN), the production of siderophores and phenol as well as chitinase activity, all metabolic factors responsible of the inhibition of *Fusarium oxysporum*, *Rhizoctonia solani,* or the nematode *Meloidogyne incognita*, just to mention some of the phytopathogens that cause high yield losses in many medicinal crops.

Very recently, endophytic fungi and bacteria identified in tea (*Camellia sinensis*) by Xie et al. [85] showed an antagonistic effect on the growth of either tea plant pathogens or pathogens of other important non-medicinal crops, such as wheat and watermelon. Other findings report the antagonistic activity of microbial endophytes of *C. sativa*, commonly known as hemp, against three of the most threatening phytopathogens, *Botrytis cinerea*, *Trichothecium roseum,* and *F. oxysporum,* the etiological agents of the “gray mold” [86], the “damping off” [87], the “pink rot” [88], and *Fusarium* wilt [89] diseased. Moreover, in this case, the hemp-associated endophytes behaved as BCA, being producers of volatile compounds, such as HCN, or other secondary metabolites, such as lytic enzymes, siderophores, and antibiotics, or displaying quorum quenching as a suitable strategy to prevent pathogen attacks hindering their growth [90]. Secondary metabolites and other bacterial elements of beneficial *Rhizobacteria* are also supposed to be involved in host induced systemic resistance (ISR) [28] or to metabolize pathogen toxins [91,92], thus reducing the pathogen toxicity or inhibiting its growth by the fungal mycelium lysis [93]. Sahu et al. [94] showed, as well, that bacterial endophytes isolated from the perennial medicinal plant, holy basil (*O. tenuiflorum*), can promote plant growth and induce ISR against *R. solani* in rice. An interesting example of the activity of the microbiota of MPs on important non-medicinal crops is given by the work of Wicaksono et al. [95,96] that described the biocontrol potential of bacterial endophytes harbored in *L. scoparium* against *Ilyonectria liriodendra*, the fungal pathogen of the grapevine, *Neofusicoccum luteum*, the fungal pathogen of the olive, and the bacterial pathogen *P. syringae* pv. actinidiae (Psa), the causal agent of bacterial canker in kiwifruit (*Actinidia deliciosa*) [96]. Moreover, these authors have shown as *L. scoparium* endophytes inoculated in the vascular system of *A. deliciosa* contribute to the protection of the host plants from Psa [95].

*L. scoparium* produces EOs; the role of EO extracted from different higher plants have been proven to be effective against soil phytopathogens without any toxic effect on the plant growth [97,98]. Since the presence of *L. scoparium* endophytes seems to modify the quality of the spectrum of *L. scoparium* EO [96], as suggested for other herbs, such as *O. vulgaris* [32] and *Thymus* spp. [33], these data could support the hypothesis of their application to the soil to reduce plant yield losses caused by soil phytopatogens. Thus, bacterial endophytes, their metabolites, and plant EOs appear to be a real valuable alternative to pesticides for sustainable agriculture both in medicinal and other important non-medicinal commercial crops.

### 3.2. Discover New Molecules to Fight Human MDR Pathogen

Human health is seriously threatened by the constant emergence of multi-drug resistant (MDR) microorganisms and the drying up of the pipelines of new antibiotic discovery. Many studies have demonstrated that bacterial endophytes of MPs could be promising sources of antimicrobial molecules [37,38,39,68,99,100]. On the other hand, since 1985, very few new classes of antibiotics have been discovered, highlighting the need for an alternative strategy to isolate effective antibiotics from endophytes [69]. Traditional use of MPs could indicate important biological factors to consider and apply in the production processes. For example, two or more antibiotic producers are often present in many traditional medicines, potentially reducing a quick resistance evolution, and the metabolomic analysis of *Aspergillus*/*Streptomyces* co-cultivation showed the production of new natural products with respect to the single culture [101]. Moreover, the antimicrobial potential of several *Streptomyces* is enhanced by the addition of other strains that produce enhancement compounds, such as cyslabadan, rather than antibiotics [102]. This molecule has been able to potentiate the β-lactams activity against methicillin-resistant *S. aureus* (MRSA). Furthermore, antimicrobial production can decline in in vitro bacteria cultivation, and possible revertant strategies are the addition of original micronutrients in the growth media and the bacterial growth inside diffusion chambers incubated in situ [103].

EOs with antimicrobial properties can be obtained from many MPs. In addition, the combination of EOs and antibiotics resulted in a synergistic effect lowering antimicrobial resistance [104]. It has been previously reported that the MP-associated microbiota is adapted to the same plant EOs [33,43,105] and the idea to use a combination of EOs and endophytic extract (from the same plant) to fight MDR pathogens is intriguing. In fact, EOs and bacterial compounds could have different modes of action on pathogens with enhanced antimicrobial effects. For instance, EOs act on the bacterial cell membrane, and a potential intracellular mechanism has been also reported [106]. Synergistic effects of an extract from endophytic *Streptomyces griseorubens* MPT42 and the EOs of the same medicinal plant (*Litsea cubeba*) have been reported with 4–16 fold reduction in MIC values when compared to the single-use against Gram-negative human pathogens [107].

Antimicrobial research deserves an important place to study the use of nanoparticles able to disrupt MDR bacterial membrane and biofilm [67,68]. In particular, metal nanoparticles obtained by biological processes result in more biocompatibility and safety than ones synthesized with traditional chemical methods. For example, the use of plant extracts for silver nanoparticle synthesis is efficient and cost-effectiveness in several different biomedical applications [108]. Reducing agents in leaves, flowers, and other parts of MPs (e.g., *A. indica*, *A. vera*, *O. basilicum*, *Emblica officinalis*, *Morinda tinctoria*, *C. roseus*, and *Justicia adhatoda*) are used to synthesize nanoparticles [109]. In this context, endophytic bacteria are also reported to produce nanoparticles [110].

**Figure 2:**
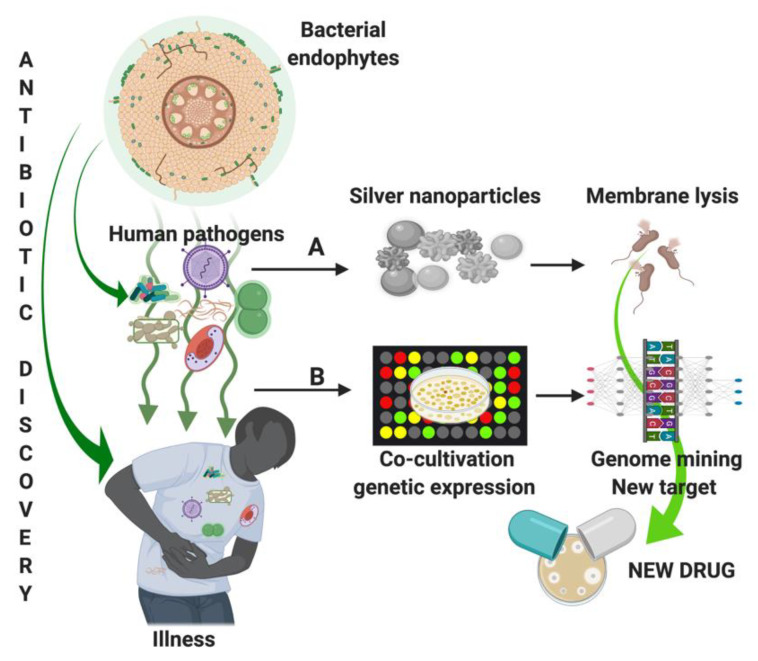
Medicinal plants’ (MP) bacterial endophytes contribute to the discovery of new antimicrobial compounds. (**A**): Endophytic biosynthesis of nanoparticles able to induce pathogen membrane lysis and DNA cleavage; (**B**): Co-cultivation of antagonistic endophytes and pathogens permits the identification of differentially expressed genes (respect to the single culture) potentially related to new targets for antibiotic treatment. Figure created using BioRender (https://biorender.com/).

Silver nanoparticles (AgNPs) synthesized by the endophytic strains of *Bacillus* spp. and fungi *Penicillium* spp. isolated from the MPs *Adhatoda beddomei*, *Curcuma longa,* and *Garcinia xanthochymus* showed an antibacterial effect on *E. coli*, *P. aeruginosa*, *S. aureus*, *S. enterica* Typhi, and *K. pneumoniae* [68,110]. Furthermore, the AgNPs synthesized by using an extract of endophytic bacterium *Pantoea ananatis* exhibited significant antimicrobial activity against *B. cereus* and *Candida albicans* resistant to conventional antibiotics (Figure 2A) [111].

Genome mining can shed light on gene clusters that remain silent under standard cultivation conditions, for example, investigating the MDR pathogen growth (and relative genetic expression) in co-cultivation with MP endophytes or on media supplemented with endophytes (Figure 2B) [99]. *Mycobacterium tuberculosis* treated with an anthraquinone molecule isolated from the mangrove endophytic fungus *Nigrospora* sp. has shown a significantly different genetic expression profile (119 out of 3875 genes) with respect to the untreated bacteria [112]. The functions of these differentially expressed genes include multiple biological processes potentially associated with new targets of anti-*M. tuberculosis* treatment. Finally, the research is investigating other approaches related to antibiotic discovery, such as genetical modulation of the specific biosynthetic or regulatory pathways and chromatin alteration [99].

The endophytic *S. coelicolor* isolated from the roots of the neem plant (*A. indica*) has been treated with the epigenetic modulator 5-azacytidine, and the bacterial extract has been tested against five human pathogenic bacteria [113]. Untreated and treated culture extracts have been proven effective against *A. hydrophila*, *S. enterica* Typhi, and *Shigella flexneri*. On the contrary, *Enterococcus faecalis* and *S. aureus* are inhibited only by the endophytic extract obtained from bacterial cultures treated with azacytidine.

Thus, epigenetic activation of the antibacterial activity of endophytic strains isolated from MPs can aid the identification of further bioactive molecules able to counteract MDR pathogens.

## 4. Conclusions

Here, the role of MP bacterial microbiota in the production of plant antimicrobial compounds is reported focusing on biotechnological and therapeutic applications. Undoubtedly, the study of the MP-endophyte interaction is a successful opportunity to develop sustainable methods to counteract human and plant pathogens. To this purpose, further development of in vitro plant–endophyte models is, therefore, needed. In fact, only the microbiota of several crops has been studied in detail for the interaction with their respective hosts, while there is a general lack of MP model systems. Recently, we proposed an in vitro model based on the interaction between the medicinal plant *E. purpurea* and its microbiota to connect fundamental and applied research on plant secondary metabolite modulation [114]. We are confident that this model and other ones developed in the same way can represent a valuable tool to discover new strategies in pathogen control.

## Data Availability

No new data were created or analyzed in this study. Data sharing is not applicable to this article. Figures were generated in Biorender (©Biorender—biorender.com); graphical abstract was adapted from “Flow chart”, by BioRender.com (2020) https://app.biorender.com/biorender-templates.

## Supplementary Materials

The following are available online at https://www.mdpi.com/2076-0817/10/2/106/s1, Table S1: Papers regarding bacterial endophytes of medicinal plants. Only works written in English in 2015–2020 are listed (last update: May 2020).

## References

[1] Firenzuoli F., Gori L. (2007). Herbal medicine today: Clinical and research issues. Evid. Based Complement. Altern. Med..

[2] Williamson E.M. (2001). Synergy and other interactions in phytomedicines. Phytomedicine.

[3] Firenzuoli F., Gori L., Neri D. (2005). Clinical phytotherapy: Opportunities and problematics. Ann. Ist. Super. Sanita.

[4] Kharwar R.N., Sharma V.K., Mishra A., Kumar J., Singh D.K., Verma S.K., Gond S.K., Kumar A., Kaushik N., Revuru B. (2020). Harnessing the phytotherapeutic treasure troves of the ancient medicinal plant azadirachta indica (Neem) and associated endophytic microorganisms. Planta Med..

[5] Jalaluddin M., Rajasekaran U.B., Paul S., Dhanya R.S., Sudeep C.B., Adarsh V.J. (2017). Comparative evaluation of neem mouthwash on plaque and gingivitis: A double-blind crossover study. J. Contemp. Dent. Pract..

[6] European Medicines Agency Recommends New Malaria Treatment for Approval. https://www.ema.europa.eu/en/news/european-medicines-agency-recommends-new-malaria-treatment-approval.

[7] Arora M., Saxena P., Choudhary D.K., Abdin M.Z., Varma A. (2016). Dual symbiosis between *Piriformospora indica* and *Azotobacter chroococcum* enhances the artemisinin content in *Artemisia annua* L. World J. Microbiol. Biotechnol..

[8] Li J., Zhao G.Z., Varma A., Qin S., Xiong Z., Huang H.Y., Zhu W.Y., Zhao L.X., Xu L.H., Zhang S. (2012). An endophytic *Pseudonocardia* species induces the production of Artemisinin in *Artemisia annua*. PLoS ONE.

[9] Nardoni S., Mugnaini L., Pistelli L., Leonardi M., Sanna V., Perrucci S., Pisseri F., Mancianti F. (2014). Clinical and mycological evaluation of an herbal antifungal formulation in canine Malassezia dermatitis. J. Mycol. Med..

[10] Perić A., Gaćeša D., Barać A., Sotirović J., Perić A.V. (2020). Herbal Drug EPs 7630 versus Amoxicillin in Patients with Uncomplicated Acute Bacterial Rhinosinusitis: A Randomized, Open-Label Study. Ann. Otol. Rhinol. Laryngol..

[11] Xie Q., Johnson B.R., Wenckus C.S., Fayad M.I., Wu C.D. (2012). Efficacy of berberine, an antimicrobial plant alkaloid, as an endodontic irrigant against a mixed-culture biofilm in an in vitro tooth model. J. Endod..

[12] Liu T., Zhang X., Tian S., Chen L., Yuan J. (2020). li Bioinformatics analysis of endophytic bacteria related to berberine in the Chinese medicinal plant Coptis teeta Wall. 3 Biotech.

[13] Maggini V., De Leo M., Mengoni A., Gallo E.R., Miceli E., Reidel R.V.B., Biffi S., Pistelli L., Fani R., Firenzuoli F. (2017). Plant-endophytes interaction influences the secondary metabolism in *Echinacea purpurea* (L.) Moench: An in vitro model. Sci. Rep..

[14] Maggini V., De Leo M., Granchi C., Tuccinardi T., Mengoni A., Gallo E.R., Biffi S., Fani R., Pistelli L., Firenzuoli F. (2019). The influence of Echinacea purpurea leaf microbiota on chicoric acid level. Sci. Rep..

[15] Abdel-Naby Awad O.G. (2020). Echinacea can help with Azithromycin in prevention of recurrent tonsillitis in children. Am. J. Otolaryngol. Head Neck Med. Surg..

[16] Isbaniah F., Wiyono W.H., Yunus F., Setiawati A., Totzke U., Verbruggen M.A. (2011). Echinacea purpurea along with zinc, selenium and vitamin C to alleviate exacerbations of chronic obstructive pulmonary disease: Results from a randomized controlled trial. J. Clin. Pharm. Ther..

[17] Wicaksono W.A., Jones E.E., Monk J., Ridgway H.J. (2016). The Bacterial Signature of Leptospermum scoparium (Mānuka) Reveals Core and Accessory Communities with Bioactive Properties. PLoS ONE.

[18] Ooi M.L., Jothin A., Bennett C., Ooi E.H., Vreugde S., Psaltis A.J., Wormald P.J. (2019). Manuka honey sinus irrigations in recalcitrant chronic rhinosinusitis: Phase 1 randomized, single-blinded, placebo-controlled trial. Int. Forum Allergy Rhinol..

[19] Compant S., Samad A., Faist H., Sessitsch A. (2019). A review on the plant microbiome: Ecology, functions, and emerging trends in microbial application. J. Adv. Res..

[20] Simon J.C., Marchesi J.R., Mougel C., Selosse M.A. (2019). Host-microbiota interactions: From holobiont theory to analysis. Microbiome.

[21] Sessitsch A., Pfaffenbichler N., Mitter B. (2019). Microbiome Applications from Lab to Field: Facing Complexity. Trends Plant Sci..

[22] Mengoni A., Pini F., Huang L.N., Shu W.S., Bazzicalupo M. (2009). Plant-by-plant variations of bacterial communities associated with leaves of the nickel hyperaccumulator Alyssum bertolonii desv. Microb. Ecol..

[23] Mengoni A., Schat H., Vangronsveld J. (2010). Plants as extreme environments? Ni-resistant bacteria and Ni-hyperaccumulators of serpentine flora. Plant Soil.

[24] Thijs S., Sillen W., Rineau F., Weyens N., Vangronsveld J. (2016). Towards an enhanced understanding of plant-microbiome interactions to improve phytoremediation: Engineering the metaorganism. Front. Microbiol..

[25] Müller D.B., Vogel C., Bai Y., Vorholt J.A. (2016). The Plant Microbiota: Systems-Level Insights and Perspectives. Annu. Rev. Genet..

[26] Green J.L., Bohannan B.J.M., Whitaker R.J. (2008). Microbial biogeography: From taxonomy to traits. Science.

[27] Tan Y., Cui Y., Li H., Kuang A., Li X., Wei Y., Xiuling J.I. (2017). Diversity and composition of rhizospheric soil and root endogenous bacteria in Panax notoginseng during continuous cropping practices. J. Basic Microbiol..

[28] Köberl M., Schmidt R., Ramadan E.M., Bauer R., Berg G. (2013). The microbiome of medicinal plants: Diversity and importance for plant growth, quality, and health. Front. Microbiol..

[29] Rodriguez R.J., Henson J., Van Volkenburgh E., Hoy M., Wright L., Beckwith F., Kim Y.O., Redman R.S. (2008). Stress tolerance in plants via habitat-adapted symbiosis. ISME J..

[30] Wardle D.A. (2006). The influence of biotic interactions on soil biodiversity. Ecol. Lett..

[31] Mengoni A., Maida I., Chiellini C., Emiliani G., Mocali S., Fabiani A., Fondi M., Firenzuoli F., Fani R. (2014). Antibiotic resistance differentiates Echinacea purpurea endophytic bacterial communities with respect to plant organs. Res. Microbiol..

[32] Pontonio E., Di Cagno R., Tarraf W., Filannino P., De Mastro G., Gobbetti M. (2018). Dynamic and Assembly of Epiphyte and Endophyte Lactic Acid Bacteria During the Life Cycle of *Origanum vulgare* L. Front. Microbiol..

[33] Checcucci A., Maida I., Bacci G., Ninno C., Bilia A.R., Biffi S., Firenzuoli F., Flamini G., Fani R., Mengoni A. (2017). Is the plant-associated microbiota of Thymus spp. adapted to plant essential oil?. Res. Microbiol..

[34] Brader G., Compant S., Mitter B., Trognitz F., Sessitsch A. (2014). Metabolic potential of endophytic bacteria. Curr. Opin. Biotechnol..

[35] Kusari P., Kusari S., Eckelmann D., Zühlke S., Kayser O., Spiteller M. (2016). Cross-species biosynthesis of maytansine in Maytenus serrata. RSC Adv..

[36] Maida I., Chiellini C., Mengoni A., Bosi E., Firenzuoli F., Fondi M., Fani R. (2016). Antagonistic interactions between endophytic cultivable bacterial communities isolated from the medicinal plant Echinacea purpurea. Environ. Microbiol..

[37] Castronovo L.M., Calonico C., Ascrizzi R., Del Duca S., Delfino V., Chioccioli S., Vassallo A., Strozza I., De Leo M., Biffi S. (2020). The Cultivable Bacterial Microbiota Associated to the Medicinal Plant *Origanum vulgare* L.: From Antibiotic Resistance to Growth-Inhibitory Properties. Front. Microbiol..

[38] Chiellini C., Maida I., Maggini V., Bosi E., Mocali S., Emiliani G., Perrin E., Firenzuoli F., Mengoni A., Fani R. (2017). Preliminary data on antibacterial activity of Echinacea purpurea-associated bacterial communities against Burkholderia cepacia complex strains, opportunistic pathogens of Cystic Fibrosis patients. Microbiol. Res..

[39] Presta L., Bosi E., Fondi M., Maida I., Perrin E., Miceli E., Maggini V., Bogani P., Firenzuoli F., Di Pilato V. (2017). Phenotypic and genomic characterization of the antimicrobial producer *Rheinheimera* sp. EpRS3 isolated from the medicinal plant Echinacea purpurea: Insights into its biotechnological relevance. Res. Microbiol..

[40] Nemr R.A., Khalil M., Sarhan M.S., Abbas M., Elsawey H., Youssef H.H., Hamza M.A., Morsi A.T., El-Tahan M., Fayez M. (2020). “In situ similis” Culturing of Plant Microbiota: A Novel Simulated Environmental Method Based on Plant Leaf Blades as Nutritional Pads. Front. Microbiol..

[41] Harrison J.G., Griffin E.A. (2020). The diversity and distribution of endophytes across biomes, plant phylogeny and host tissues: How far have we come and where do we go from here?. Environ. Microbiol..

[42] Akinsanya M.A., Goh J.K., Lim S.P., Ting A.S.Y. (2015). Metagenomics study of endophytic bacteria in Aloe vera using next-generation technology. Genom. Data.

[43] Emiliani G., Mengoni A., Maida I., Perrin E., Chiellini C., Fondi M., Gallo E., Gori L., Maggini V., Vannacci A. (2014). Linking bacterial endophytic communities to essential oils: Clues from *Lavandula angustifolia* mill. Evid. Based Complement. Altern. Med..

[44] Chiellini C., Maida I., Emiliani G., Mengoni A., Mocali S., Fabiani A., Biffi S., Maggini V., Gori L., Vannacci A. (2015). Endophytic and rhizospheric bacterial communities isolated from the medicinal plants echinacea purpurea and echinacea angustifolia. Int. Microbiol..

[45] Strobel G.A. (2003). Endophytes as sources of bioactive products. Microbes Infect..

[46] Jasim B., Sreelakshmi S., Mathew J., Radhakrishnan E.K. (2016). Identification of endophytic Bacillus mojavensis with highly specialized broad spectrum antibacterial activity. 3 Biotech.

[47] Roy S., Yasmin S., Ghosh S., Bhattacharya S., Banerjee D. (2016). Anti-Infective Metabolites of a Newly Isolated Bacillus thuringiensis KL1 Associated with Kalmegh (Andrographis paniculata Nees.), a Traditional Medicinal Herb. Microbiol. Insights.

[48] Castillo U.F., Strobel G.A., Ford E.J., Hess W.M., Porter H., Jensen J.B., Albert H., Robison R., Condron M.A.M., Teplow D.B. (2002). Munumbicins, wide-spectrum antibiotics produced by Streptomyces NRRL 30562, endophytic on Kennedia nigriscans. Microbiology.

[49] Conti R., Chagas F.O., Caraballo-Rodriguez A.M., da Paixão Melo W.G., do Nascimento A.M., Cavalcanti B.C., de Moraes M.O., Pessoa C., Costa-Lotufo L.V., Krogh R. (2016). Endophytic Actinobacteria from the Brazilian Medicinal Plant Lychnophora ericoides Mart. and the Biological Potential of Their Secondary Metabolites. Chem. Biodivers..

[50] Taechowisan T., Chanaphat S., Ruensamran W., Phutdhawong W.S. (2012). Antifungal activity of 3-methylcarbazoles from Streptomyces sp. LJK109; an endophyte in Alpinia galanga. J. Appl. Pharm. Sci..

[51] Ludwig-Müller J. (2015). Plants and endophytes: Equal partners in secondary metabolite production?. Biotechnol. Lett..

[52] Li J., Zhao G.Z., Huang H.Y., Qin S., Zhu W.Y., Zhao L.X., Xu L.H., Zhang S., Li W.J., Strobel G. (2012). Isolation and characterization of culturable endophytic actinobacteria associated with *Artemisia annua* L. Antonie Van Leeuwenhoekint. J. Gen. Mol. Microbiol..

[53] Pandey S.S., Singh S., Babu C.S.V., Shanker K., Srivastava N.K., Kalra A. (2016). Endophytes of opium poppy differentially modulate host plant productivity and genes for the biosynthetic pathway of benzylisoquinoline alkaloids. Planta.

[54] Pandey S.S., Singh S., Pandey H., Srivastava M., Ray T., Soni S., Pandey A., Shanker K., Babu C.S.V., Banerjee S. (2018). Endophytes of Withania somnifera modulate in planta content and the site of withanolide biosynthesis. Sci. Rep..

[55] Tiwari R., Awasthi A., Mall M., Shukla A.K., Srinivas K.V.N.S., Syamasundar K.V., Kalra A. (2013). Bacterial endophyte-mediated enhancement of in planta content of key terpenoid indole alkaloids and growth parameters of Catharanthus roseus. Ind. Crop. Prod..

[56] Liu Z., Zhou J., Li Y., Wen J., Wang R. (2020). Bacterial endophytes from Lycoris radiata promote the accumulation of Amaryllidaceae alkaloids. Microbiol. Res..

[57] Egamberdieva D., Wirth S., Behrendt U., Ahmad P., Berg G. (2017). Antimicrobial Activity of Medicinal Plants Correlates with the Proportion of Antagonistic Endophytes. Front. Microbiol..

[58] Passari A.K., Leo V.V., Singh G., Samanta L., Ram H., Siddaiah C.N., Hashem A., Al-Arjani A.B.F., Alqarawi A.A., Abd_Allah E.F. (2020). In vivo studies of inoculated plants and in vitro studies utilizing methanolic extracts of endophytic Streptomyces sp. Strain dbt34 obtained from Mirabilis jalapa L. exhibit ros-scavenging and other bioactive properties. Int. J. Mol. Sci..

[59] Mishra A., Singh S.P., Mahfooz S., Bhattacharya A., Mishra N., Shirke P.A., Nautiyal C.S. (2018). Bacterial endophytes modulates the withanolide biosynthetic pathway and physiological performance in Withania somnifera under biotic stress. Microbiol. Res..

[60] Zhou J.Y., Li X., Zhao D., Deng-Wang M.Y., Dai C.C. (2016). Reactive oxygen species and hormone signaling cascades in endophytic bacterium induced essential oil accumulation in Atractylodes lancea. Planta.

[61] Zhou J.Y., Sun K., Chen F., Yuan J., Li X., Dai C.C. (2018). Endophytic Pseudomonas induces metabolic flux changes that enhance medicinal sesquiterpenoid accumulation in Atractylodes lancea. Plant Physiol. Biochem..

[62] Qin S., Feng W.W., Zhang Y.J., Wang T.T., Xiong Y.W., Xing K. (2018). Diversity of bacterial microbiota of coastal halophyte Limonium sinense and amelioration of salinity stress damage by symbiotic plant growthpromoting actinobacterium Glutamicibacter halophytocola KLBMP 5180. Appl. Environ. Microbiol..

[63] Devi K.A., Pandey G., Rawat A.K.S., Sharma G.D., Pandey P. (2017). The Endophytic Symbiont—Pseudomonas aeruginosa Stimulates the Antioxidant Activity and Growth of *Achyranthes aspera* L. Front. Microbiol..

[64] Purushotham N., Jones E., Monk J., Ridgway H. (2018). Community Structure of Endophytic Actinobacteria in a New Zealand Native Medicinal Plant Pseudowintera colorata (Horopito) and Their Influence on Plant Growth. Microb. Ecol..

[65] Ulloa-Muñoz R., Olivera-Gonzales P., Castañeda-Barreto A., Villena G.K., Tamariz-Angeles C. (2020). Diversity of endophytic plant-growth microorganisms from Gentianella weberbaueri and Valeriana pycnantha, highland Peruvian medicinal plants. Microbiol. Res..

[66] El-Sayed A.S.A., El-Sayed M.T., Rady A.M., Zein N., Enan G., Shindia A., El-Hefnawy S., Sitohy M., Sitohy B. (2020). Exploiting the Biosynthetic Potency of Taxol from Fungal Endophytes of Conifers Plants; Genome Mining and Metabolic Manipulation. Molecules.

[67] Christina A., Christapher V., Bhore S. (2013). Endophytic bacteria as a source of novel antibiotics: An overview. Pharmacogn. Rev..

[68] Singh M., Kumar A., Singh R., Pandey K.D. (2017). Endophytic bacteria: A new source of bioactive compounds. 3 Biotech.

[69] Quinn G.A., Banat A.M., Abdelhameed A.M., Banat I.M. (2020). Streptomyces from traditional medicine: Sources of new innovations in antibiotic discovery. J. Med. Microbiol..

[70] Nagpal S., Sharma P., Sirari A., Gupta R.K. (2020). Coordination of Mesorhizobium sp. and endophytic bacteria as elicitor of biocontrol against Fusarium wilt in chickpea. Eur. J. Plant Pathol..

[71] Vurukonda S.S.K.P., Giovanardi D., Stefani E. (2018). Plant growth promoting and biocontrol activity of Streptomyces spp. as endophytes. Int. J. Mol. Sci..

[72] Sundaramoorthy S. (2012). Consortial Effect of Endophytic and Plant Growth Promoting Rhizobacteria for the Management of Early Blight of Tomato Incited by Alternaria Solani. J. Plant Pathol. Microbiol..

[73] Michel-Aceves A.C., Díaz-Nájera J.F., Ariza-Flores R., Otero-Sánchez M.A., Escobar-Martínez R., Avendaño-Arrazate C.H. (2019). Control alternatives for damping-off in tomato seedling production. Phyton.

[74] Huang X., Ren J., Li P., Feng S., Dong P., Ren M. (2020). Potential of microbial endophytes to enhance the resistance to postharvest diseases of fruit and vegetables. J. Sci. Food Agric..

[75] Resti Z., Liswarni Y., Martinius M. (2020). Endophytic Bacterial Consortia as Biological Control of Bacterial Leaf Blight and Plant Growth Promoter of Rice (*Oryza sativa* L.). J. Appl. Agric. Sci. Technol..

[76] Pal K.K., McSpadden Gardener B. (2006). Biological Control of Plant Pathogens. Plant Health Instr..

[77] Lecomte C., Alabouvette C., Edel-Hermann V., Robert F., Steinberg C. (2016). Biological control of ornamental plant diseases caused by Fusarium oxysporum: A review. Biol. Control.

[78] Eljounaidi K., Lee S.K., Bae H. (2016). Bacterial endophytes as potential biocontrol agents of vascular wilt diseases—Review and future prospects. Biol. Control.

[79] Latz M.A.C., Jensen B., Collinge D.B., Jørgensen H.J.L. (2018). Endophytic fungi as biocontrol agents: Elucidating mechanisms in disease suppression. Plant Ecol. Divers..

[80] De Silva N.I., Brooks S., Lumyong S., Hyde K.D. (2019). Use of endophytes as biocontrol agents. Fungal Biol. Rev..

[81] Morales-Cedeño L.R., del Carmen Orozco-Mosqueda M., Loeza-Lara P.D., Parra-Cota F.I., de los Santos-Villalobos S., Santoyo G. (2021). Plant growth-promoting bacterial endophytes as biocontrol agents of pre- and post-harvest diseases: Fundamentals, methods of application and future perspectives. Microbiol. Res..

[82] Santoyo G., del Orozco-Mosqueda M.C., Govindappa M. (2012). Mechanisms of biocontrol and plant growth-promoting activity in soil bacterial species of Bacillus and Pseudomonas: A review. Biocontrol Sci. Technol..

[83] Ahmed E.A., Hassan E.A., Tobgy K.M.K.E., Ramadan E.M. (2014). Evaluation of rhizobacteria of some medicinal plants for plant growth promotion and biological control. Ann. Agric. Sci..

[84] Singh S.K., Pathak R. (2015). Ecological Manipulations of Rhizobacteria for Curbing Medicinal Plant Diseases.

[85] Xie H., Feng X., Wang M., Wang Y., Kumar Awasthi M., Xu P. (2020). Implications of endophytic microbiota in *Camellia sinensis*: A review on current understanding and future insights. Bioengineered.

[86] Barloy J., Pelhate J. (1962). Premieres observations phytopathologiques relatives aux cultures de chanvre en Anjou. Ann. Epiphyt..

[87] Doctor B. (1985). Damping off. Sinsemilla Tips.

[88] McPartland J.M. (1991). Common names for diseases of *Cannabis sativa* L. Plant Dis..

[89] Snyder W.C., Hansen H.N. (1940). The Species Concept in Fusarium. Am. J. Bot..

[90] Taghinasab M., Jabaji S. (2020). Cannabis Microbiome and the Role of Endophytes in Modulating the Production of Secondary Metabolites: An Overview. Microorganisms.

[91] Toyoda H., Utsumi R. (1991). Method for the Prevention of Fusarium Diseases and Microorganisms Used for the Same. U.S. Patent.

[92] Simonetti E., Roberts I.N., Montecchia M.S., Gutierrez-Boem F.H., Gomez F.M., Ruiz J.A. (2018). A novel Burkholderia ambifaria strain able to degrade the mycotoxin fusaric acid and to inhibit Fusarium spp. growth. Microbiol. Res..

[93] Mauch F., Mauch-Mani B., Boller T. (1988). Antifungal Hydrolases in Pea Tissue. Plant Physiol..

[94] Sahu P.K., Singh S., Gupta A.R., Gupta A., Singh U.B., Manzar N., Bhowmik A., Singh H.V., Saxena A.K. (2020). Endophytic bacilli from medicinal-aromatic perennial Holy basil (*Ocimum tenuiflorum* L.) modulate plant growth promotion and induced systemic resistance against Rhizoctonia solani in rice (*Oryza sativa* L.). Biol. Control.

[95] Wicaksono W.A., Jones E.E., Casonato S., Monk J., Ridgway H.J. (2018). Biological control of Pseudomonas syringae pv. actinidiae (Psa), the causal agent of bacterial canker of kiwifruit, using endophytic bacteria recovered from a medicinal plant. Biol. Control.

[96] Wicaksono W.A., Jones E.E., Sansom C.E., Perry N.B., Monk J., Black A., Ridgway H.J. (2017). Indigenous bacteria enhance growth and modify essential oil content in Leptospermum scoparium (mānuka). N. Z. J. Bot..

[97] Raveau R., Fontaine J., Lounès-Hadj Sahraoui A. (2020). Essential oils as potential alternative biocontrol products against plant pathogens and weeds: A review. Foods.

[98] Pandey V.N., Dubey N.K. (1994). Antifungal potential of leaves and essential oils from higher plants against soil phytopathogens. Soil Biol. Biochem..

[99] Kealey C., Creaven C.A., Murphy C.D., Brady C.B. (2017). New approaches to antibiotic discovery. Biotechnol. Lett..

[100] Martinez-Klimova E., Rodríguez-Peña K., Sánchez S. (2017). Endophytes as sources of antibiotics. Biochem. Pharmacol..

[101] Wu C., Zacchetti B., Ram A.F.J., Van Wezel G.P., Claessen D., Choi Y.H. (2015). Expanding the chemical space for natural products by Aspergillus-Streptomyces co-cultivation and biotransformation. Sci. Rep..

[102] Fukumoto A., Kim Y.P., Matsumoto A., Takahashi Y., Shiomi K., Tomoda H., Omura S. (2008). Cyslabdan, a new potentiator of imipenem activity against methicillin-resistant Staphylococcus aureus, produced by Streptomyces sp. K04-0144: I. Taxonomy, fermentation, isolation and structural elucidation. J. Antibiot..

[103] Nichols D., Cahoon N., Trakhtenberg E.M., Pham L., Mehta A., Belanger A., Kanigan T., Lewis K., Epstein S.S. (2010). Use of ichip for high-throughput in situ cultivation of “uncultivable microbial species”. Appl. Environ. Microbiol..

[104] Yap P.S.X., Krishnan T., Yiap B.C., Hu C.P., Chan K.G., Lim S.H.E. (2014). Membrane disruption and anti-quorum sensing effects of synergistic interaction between Lavandula angustifolia (lavender oil) in combination with antibiotic against plasmid-conferred multi-drug-resistant Escherichia coli. J. Appl. Microbiol..

[105] Da Silva T.F., Vollú R.E., Jurelevicius D., Alviano D.S., Alviano C.S., Blank A.F., Seldin L. (2013). Does the essential oil of Lippia sidoides Cham. (Pepper-rosmarin) affect its endophytic microbial community?. Bmc Microbiol..

[106] Perrin E., Maggini V., Maida I., Gallo E., Lombardo K., Madarena M.P., Buroni S., Scoffone V.C., Firenzuoli F., Mengoni A. (2018). Antimicrobial activity of six essential oils against Burkholderia cepacia complex: Insights into mechanism(s) of action. Future Microbiol..

[107] Nguyen Q.H., Van Nguyen H., Vu T.H.N., Chu-Ky S., Vu T.T., Hoang H., Quach N.T., Bui T.L., Chu H.H., Khieu T.N. (2019). Characterization of endophytic Streptomyces griseorubens MPT42 and assessment of antimicrobial synergistic interactions of its extract and essential oil from host plant Litsea cubeba. Antibiotics.

[108] Bose D., Chatterjee S. (2015). Antibacterial Activity of Green Synthesized Silver Nanoparticles Using Vasaka (*Justicia adhatoda* L.) Leaf Extract. Indian J. Microbiol..

[109] Thirumagal N., Jeyakumari A.P. (2020). Structural, Optical and Antibacterial Properties of Green Synthesized Silver Nanoparticles (AgNPs) Using *Justicia adhatoda* L. Leaf Extract. J. Clust. Sci..

[110] Sunkar S., Nachiyar C.V. (2012). Biogenesis of antibacterial silver nanoparticles using the endophytic bacterium Bacillus cereus isolated from Garcinia xanthochymus. Asian Pac. J. Trop. Biomed..

[111] Monowar T., Rahman S., Bhore S.J., Raju G., Sathasivam K.V. (2018). Silver Nanoparticles Synthesized by Using the Endophytic Bacterium Pantoea ananatis are Promising Antimicrobial Agents against Multidrug Resistant Bacteria. Molecules.

[112] Wang C., Wang J., Huang Y., Chen H., Li Y., Zhong L., Chen Y., Chen S., Wang J., Kang J. (2013). Anti-mycobacterial activity of marine fungus-derived 4-deoxybostrycin and nigrosporin. Molecules.

[113] Kumar J., Sharma V.K., Singh D.K., Mishra A., Gond S.K., Verma S.K., Kumar A., Kharwar R.N. (2016). Epigenetic Activation of Antibacterial Property of an Endophytic Streptomyces coelicolor Strain AZRA 37 and Identification of the Induced Protein Using MALDI TOF MS/MS. PLoS ONE.

[114] Maggini V., Mengoni A., Bogani P., Firenzuoli F., Fani R. (2020). Promoting Model Systems of Microbiota–Medicinal Plant Interactions. Trends Plant Sci..

